# Deciphering plant health status: The link between secondary metabolites, fungal community and disease incidence in olive tree

**DOI:** 10.3389/fpls.2023.1048762

**Published:** 2023-03-22

**Authors:** Teresa Gomes, José Alberto Pereira, Jordi Moya-Laraño, Jorge Poveda, Teresa Lino-Neto, Paula Baptista

**Affiliations:** ^1^ Centro De Investigação De Montanha (CIMO), Instituto Politécnico de Bragança, Bragança, Portugal; ^2^ Laboratório Associado Para a Sustentabilidade e Tecnologia Em Regiões De Montanha (SusTEC), Instituto Politécnico De Bragança, Bragança, Portugal; ^3^ Functional and Evolutionary Ecology, Estación Experimental De Zonas Áridas - CSIC, Almería, Spain; ^4^ Institute for Multidisciplinary Research in Applied Biology (IMAB), Universidad Pública De Navarra, Pamplona, Spain; ^5^ Centre of Molecular and Environmental Biology (CBMA), Department of Biology, University of Minho, Braga, Portugal

**Keywords:** *Venturia oleaginea*, epiphytes, endophytes, volatile compounds, phenolic compounds

## Abstract

Plant-associated microorganisms are increasingly recognized to play key roles in host health. Among several strategies, associated microorganisms can promote the production of specific metabolites by their hosts. However, there is still a huge gap in the understanding of such mechanisms in plant-microorganism interaction. Here, we want to determine whether different levels of olive leaf spot (OLS) disease incidence were related to differences in the composition of fungal and secondary metabolites (*i.e.* phenolic and volatile compounds) in leaves from olive tree cultivars with contrasting OLS susceptibilities (ranging from tolerant to highly susceptible). Accordingly, leaves with three levels of OLS incidence from both cultivars were used to assess epiphytic and endophytic fungal communities, by barcoding of cultivable isolates, as well as to evaluate leaf phenolic and volatile composition. Fungal and metabolite compositions variations were detected according to the level of disease incidence. Changes were particularly noticed for OLS-tolerant cultivars, opposing to OLS-susceptible cultivars, suggesting that disease development is linked, not only to leaf fungal and metabolite composition, but also to host genotype. A set of metabolites/fungi that can act as predictive biomarkers of plant tolerance/susceptibility to OLS disease were identified. The metabolites α-farnesene and p-cymene, and the fungi *Fusarium* sp. and *Alternaria* sp. were more related to disease incidence, while *Pyronema domesticum* was related to the absence of disease symptoms. Cultivar susceptibility to OLS disease is then suggested to be driven by fungi, volatile and phenolic host leaves composition, and above all to plant-fungus interaction. A deeper understanding of these complex interactions may unravel plant defensive responses.

## 1 Introduction

In natural ecosystems, the aboveground parts of plants come across a myriad of fungal species that can colonize the surface (epiphytes) or internal plant tissues (endophytes), with beneficial or detrimental outcomes (Zeilinger et al., 2016). For example, different fungal groups (e.g. *Trichoderma* and *Epicoccum*) have demonstrated the ability to protect host plants from pathogens, while others (e.g. *Colletotrichum*) are well-recognized pathogens causing plant diseases (Poveda and Baptista, 2021). Despite the ecological and agricultural importance of plant-fungal associations, the complex interaction network occurring among fungi and plants is still not fully understood (Chaudhry et al., 2021). There are still many open questions that remain unanswered regarding how plant-associated microorganisms contribute to the health status of their host. Recent studies have provided strong evidences about the capacity of endophytes to improve plant protection against pathogens by supplying several bioactive metabolites to their host (Fadiji and Babalola, 2020). From the wide range of secondary metabolites that are induced during plant-microbial interactions, both phenolic and volatile organic compounds (VOCs) seem to be particularly important, due to their recognized antimicrobial activity and ability to induce plant defenses against pathogens (Tilocca et al., 2020; Wallis and Galarneau, 2020; Poveda, 2021). However, the elucidation of plant associated microorganism potential to improve plant health through the synthesis of bioactive metabolites in host tissues is a challenging task, especially if studied in the nature. In fact, the microorganisms that interact with plants are ubiquitous in nature and can contribute to metabolite production in different ways. Microorganisms can produce their own secondary metabolites (which will be mixed with those produced by plant host), change the biosynthesis of plant host metabolites, or even metabolize plant host secondary metabolites and produce new metabolites (Pang et al., 2021). Probably due to such complex aspects occurring during plant-microorganism interactions, few studies have focused on the mechanisms employed by microorganisms in protecting host plant from pathogens.

The olive leaf spot (OLS) disease, caused by the fungus *Venturia oleaginea* (Castagne) Rossman & Crous (syn. *Fusicladium oleagineum*, *Spilocaea oleaginea*), is one of the most damaging diseases of olive tree (*Olea europaea* L.) worldwide (Viruega et al., 2013). Fungal development is mostly restricted to olive leaf tissues, including leaf surface and subcuticular areas, causing scab lesions and leaf-drop symptoms, leading occasionally to tree death (Viruega et al., 2013). Under the same agro-climatic conditions, the OLS disease is more severe in certain olive tree cultivars (*e.g*. “Madural” and “Verdeal Transmontana”) than in others (*e.g*. “Cobrançosa*”*) (Pereira, J.A., Per. Com.). In the present work, this biological system was chosen as a model for studying the impact of interactions occurring among fungi and plant hosts on the plant health status. Indeed, the availability of olive cultivars with distinct susceptibility levels to OLS, and with the possibility in displaying different levels of disease incidence, is an advantage. Detected differences on fungal communities or metabolites (volatile and phenolic compounds) of host plant leaves could thus be linked to the cultivar or disease incidence effect. Moreover, using this model, the simultaneous study of interactions occurring between plant and epiphytes or endophytes is possible. This is particularly relevant due to the recognized ability of *V. oleaginea* to develop in the surface and subcuticular spaces of the leaves. By considering these aspects, we hypothesized that plant interactions with fungi could modify plant secondary metabolites composition, thus affecting the incidence of OLS disease on these cultivars. Specifically, we address the following questions: (1) Is OLS incidence related to host-associated epiphytic and endophytic fungal communities composition in leaves? (2) Is OLS incidence related to host plant composition on phenolic and volatile compounds? (3) Is there any relation among fungal consortia and secondary metabolites composition that could explain different incidence levels of OLS disease? As far as we known, no previous investigation has addressed concerning fungal communities and chemical composition of leaves as a whole. The understanding of these complex associations (i.e. host plant, phytochemicals, fungal communities and disease incidence) might improve our knowledge on the role of different fungal taxa and metabolites in OLS disease incidence.

## 2 Materials and methods

### 2.1 Study site and olive leaves collection

The study was conducted in two olive orchards at Mirandela region (Northeast of Portugal), at coordinates N 41° 32.593’ W 07° 07.445’ (orchard 1) and N 41° 29.490’ W 07° 15.413’ (orchard 2). Each orchard comprises olive trees from three olive cultivars, *i.e*. “Cobrançosa”, “Madural” and “Verdeal Transmontana”, at the spacing of 7 x 7m, and is managed through integrated production guidelines (Malavolta and Perdikis, 2018). These three cultivars are considered tolerant (“Cobrançosa”) and susceptible (“Madural” and “Verdeal Transmontana”) to OLS disease. In each orchard, five trees per cultivar were randomly selected in close proximity to each other. Leaves were randomly collected in the four orientations of the tree canopy, at 1.5 meters above the ground, from March to May. The collected leaves were used to assess OLS disease incidence of each tree (% infected leaves), to determine epiphytic and endophytic fungal communities, as well as chemical composition (*i.e*., phenolic and volatile compounds). The disease incidence (%) in each surveyed olive tree was assessed using a total of 60 randomly collected leaves. The number of observed symptomatic leaves was used for determining the percentage of infected leaves. For chemical evaluations, and to mimic natural conditions within the tree canopy, a mixture of ten randomly selected leaves per tree was used, comprising five leaves with visible spots (OLS-symptomatic leaves) and five leaves without visible spots (asymptomatic leaves). For fungal diversity assessment was used a similar procedure by using a mixture of six leaves per tree (three OLS-symptomatic leaves and three asymptomatic leaves). All evaluations were performed using fresh leaves (immediately upon their collection), with exception of assessment of phenolic compounds that used lyophilized leaves. For this, leaves were stored in a deep freezer at -20°C, lyophilized, ground to a fine powder using an analytical mill, and stored in a dark room until phenolic analysis.

### 2.2 Assessment of foliar fungal communities

#### 2.2.1 Fungal isolation

Both epiphytic and endophytic fungal communities in olive tree leaves were evaluated based on culture-dependent methods. The isolation of fungal epiphytes was performed by the dilution plate method, by using around 1-gram weight of leaf samples in 9 mL of sterile potassium phosphate buffer pH 7.0 (0.20 g/L KCl; 8 g/L NaCl; 1.4 g/L Na_2_HPO_4_; 0.24 g/L KH_2_PO_4_), according to the procedure described by Gomes et al. (2018). Briefly, aliquots (1 ml) of the resulting microbial suspension were separately plated in triplicate onto Potato Dextrose Agar (PDA, Difco) and Plate Count Agar (PCA, Himedia) media, supplemented with 0.01% (w/v) chloramphenicol (Oxoid, Basingstoke, Hampshire, UK). In total, 1,080 Petri plates were inoculated (30 trees x 6 leaves x 2 culture media x 3 replicates). Plates were incubated at 25 ± 2°C in the dark for fungal growth and colonies counting. The number of epiphytes (i.e. the number of individual colonies of fungi on the leaf surface) was expressed as log CFU/cm^2^. For estimating the leaf surface, an ellipse equation (A=πab) was used, being A the area, whereas a and b were the half-length of longitudinal and transverse axes of a leaf, respectively.

Endophytic fungi were isolated from the same leaves used to isolate epiphytes. Epiphytes were removed by surface disinfection of leaves, using the procedure previously optimized by Martins et al. (2016), which consisted in the sequential immersion of leaves in 70% (v/v) ethanol for 2 min, 3–5% (v/v) sodium hypochlorite for 3 min, 70% (v/v) ethanol for 1 min, and sterile distilled water (three times, 1 min each). After disinfection, each leaf was cut into five fragments (ca. 5 x 5 mm), which were transferred to the same culture media used to isolate epiphytes. Endophytic fungi were isolated from a total of 1,800 leaf tissue segments (30 trees x 6 leaves x 2 culture media x 5 fragments). Validation of the surface sterilization procedure was done by imprinting the surface of sterilized leaf tissues onto PDA and PCA media. Emerging fungal colonies were subcultured on fresh medium until pure epiphytic/endophytic cultures were obtained.

#### 2.2.2 Fungal identification

Each fungal colony was identified by using morphological and molecular approaches, according to Gomes et al. (2018). Briefly, fungal isolates were firstly grouped according to their morphological similarity at colony level (colony appearances, mycelial textures, spore mass color, diffusible pigment and pigmentations on both obverse and reverse of colonies). Three representative isolates of each morphotype were further selected for molecular identification, using the internal transcribed spacer (ITS) region of nuclear ribosomal DNA (rDNA). Total genomic DNA was extracted from harvested mycelial/spores using the REDExtract-N-Amp™ Plant PCR kit (Sigma, Poole, UK) following manufacturer’s instructions. The ITS region (ITS1, 5.8S, ITS2) was amplified using ITS1/ITS4 primers set (White et al., 1990). Amplifications occurred in a MyCycler™ (Bio-Rad) thermocycler, using 50 µL PCR reactions, which contained 5 µL of 10x complete PCR buffer (0.1% tween 20, 25 mM MgCl_2_, pH 8.8), 1 µL dNTPs of 10 mM, 1 µL of each primer (10 µM), 4 µL of DNA, 0.2 µL of DFS-Taq DNA Polymerase (5 U/µL) (BIORON GmbH) and 37.8 µL of distilled sterile water. The PCR program was set for an initial denaturation step at 95°C for 5min, followed by 30 cycles of denaturation at 94°C for 40s, primer annealing at 48°C – 56°C (being 54°C the most used) for 50s and extension at 72°C for 45s, followed by a final extension step at 72°C for 7 min. The amplified products (~ 650 bp) were purified and sequenced using Macrogen Inc. (Madrid, Spain) services. The obtained DNA sequences were analysed with DNASTAR v.2.58 software and fungal identification was performed using the NCBI database (http://www.ncbi.nlm.nih.gov) and BLAST algorithm, according the procedure described by Gomes et al. (2018). The obtained sequences are available at GenBank with the following accession numbers: KU324941-KU325040; KU325041-KU325240; KU325241-KU325457. Each operational taxonomic unit (OTU) was taxonomically classified according to the Index Fungorum Database (www.indexfungorum.org).

### 2.3 Phenolic compounds identification and quantification

#### 2.3.1 Standards and reagents

Used standards were purchased from Sigma (St. Louis, MO, USA) or Extrasynthèse (Genay, France). Methanol and formic acid were obtained from Merck (Darmstadt, Germany). The water was treated in a Milli-Q water purification system (Millipore, Bedford, MA, USA) before use.

#### 2.3.2 Extraction of phenolic compounds

Before the extraction of phenolic compounds, each lyophilized leaf sample was powered and sieved using a 900 μm sieve. The extraction was performed as previously described by Vinha et al. (2002). Briefly, about 1.5 g of the powdered leaf samples were weighed in quadruplicates. Each sample was separately mixed with 50 mL of methanol (99.96%, Aldrich) at 150 rpm for 1 h (room temperature). The obtained methanolic extracts were filtered through a Whatman No.4 paper and evaporated (Stuart RE3000, UK) to dryness under reduced pressure (35°C). After dissolution in 2 ml methanol (99.96%, Aldrich) and filtration (Whatman No. 2), an aliquot of 20 μl of the obtained extracts was analyzed by HPLC.

#### 2.3.3 Analysis of phenolic compounds

Chromatographic separation was performed as previously reported by Vinha et al. (2002), with an analytical HPLC unit (Knauer Smartline), equipped with a Knauer Smartline autosampler 3800, and a Knauer Diode Array Detector (DAD). A reversed-phase Spherisorb ODS2 column was used during analysis (250 x 4.6 mm, 5 μm particle size, Merck, Darmstadt, Germany). The used solvent system was a gradient of water–formic acid (19:1) and methanol, applied at a flow rate of 0.9 mL min^−1^. Spectral data from all peaks were accumulated within the 200–400 nm range. Chromatograms were recorded at 280, 320 and 350 nm, and data were managed on ClarityChrom^®^ software (Knauer, Berlin, Germany). Phenolic compounds were quantified through the comparison performed with known amounts of external standards: hydroxytyrosol, oleuropein, chlorogenic acid and rutin were quantified at 280 nm, caffeic acid at 320 nm, and verbascoside, apigenin-7-O-glucoside, luteolin-7-O-glucoside and luteolin at 350 nm. HPLC analyses were performed using two technical replicates for each extract. The means of the four replicates for each tree leaf sample were then calculated. Phenolic compounds were expressed as the amount of phenolics per dry weight (DW) of leaf extract (mg/g of DW).

### 2.4 Volatile identification and quantification

The extraction and analysis of volatile compounds from fresh leaves were performed according to the methodology described by Malheiro et al. (2015), with some modifications.

#### 2.4.1 Extraction of volatile compounds

The extraction of leaf volatiles was performed by headspace solid phase microextraction (HS-SPME). Around 1-gram weight of fresh leaves was placed in 50 ml vials, containing 10 μl of 4-methyl 2-pentanol (10.65 ppm dissolved in methanol), which was used as an internal standard. The vials were sealed with a polypropylene cap with a silicon septum. Following an incubation in an ultrasonic bath at 40°C, for 10 min, the divinylbenzene/carboxen/polydimethylsiloxane (DVB/CAR/PDMS; 50/30 μm) fiber was inserted into the vial headspace for more than 30 minutes, at 40°C, for volatile adsorption. The volatiles were desorbed by placing the fiber into the gas chromatographic (GC) injection port for 10 min, at 280°C. The HS-SPME analyses were performed using five replicates for each tree leaf sample.

#### 2.4.2 Gas chromatography-mass spectrometry (GC-MS) conditions

Chromatographic analysis was performed on an Agilent 6890 series GC (Agilent, Avondale, PA, USA), with splitless injection, coupled to a MS detector (Agilent 5973), according to the conditions described by Malheiro et al. (2015). Volatile compounds were identified by comparing the experimental spectra with spectra from NIST data bank (NIST/EPA/NISH Mass Spectral Library, version 1.6, U.S.A.) and also by comparison of their GC retention index. Retention indices were determined as reported by Malheiro et al. (2015). Concentration of identified compounds were calculated by the ratio of each individual base ion peak area to the area of the internal standard. The obtained ratio was then converted to mass equivalents, on the basis on the internal mass standard added. Volatiles were represented as the amount of volatile compound per fresh weight (FW) of leaf tissue (mg/kg of FW).

### 2.5 Data analysis

Based on OLS disease assessment results, three ranges of disease incidence were defined: 0-5%, 5-10%, and 10-15%. Data was analyzed considering each group of disease incidence and each cultivar. Thus, a total of nine experimental units were established (three ranges of disease incidence per cultivar), each one with a sample size of three to four trees. The normality assumption of the data was verified using the Shapiro-Wilk test.

#### 2.5.1 Differences on fungal communities and metabolite profiles among OLS incidence ranges and cultivars

The total number and abundance of fungal OTUs and metabolites (phenolic and volatiles compounds) for each olive tree are presented as the mean for each OLS disease incidence range (0-5%, 5-10%, 10-15%) and cultivar (“Cobrançosa”, “Madural”, “Verdeal Transmontana”). Differences between means were evaluated by one-way analysis of variance (One-way ANOVA) with SPSS v.20, followed by Tukey’s *post hoc* test (p <0.05). Non-metric Multidimensional Scaling (NMDS) plots, based on Bray–Curtis distances, were performed to assess the variation in the composition of foliar fungal communities and metabolite profiles, among different ranges of OLS disease incidence (0-5%, 5-10%, 10-15%). Kruskal’s stress was used to estimate goodness of fit (commonly acceptable when <0.2). A one-way analysis of similarity (ANOSIM) was used to determine significant differences in fungal (or metabolite) compositions among OLS incidence groupings, using Bray–Curtis distance matrices. ANOSIM generates a P-value (significant level below to 0.05) and a R-value, which gives the degree of discrimination between groups and ranges from 0 (indistinguishable) to 1 (completely dissimilar) (Clarke and Gorley, 2015). NMDS plots and ANOSIM analyses were performed using *Community Analysis Package* v. 6.0 (Henderson and Seaby, 2019). Subsequent analyses were performed in R (R Core Team 2018). Using the ‘heatmap 2’ function of *gplots* package, with the Euclidean distance, heatmaps with hierarchical clustering were constructed for grouping host cultivars and OLS incidence ranges, according to the abundance of fungal OTUs (abundance >10) and metabolites (abundance >12). Each sample was transformed into a row Z-score and high relative values were colored differently from those with low relative values.

#### 2.5.2 Relationship between OLS disease incidence, host cultivar, fungal OTUs and metabolites

Random forest analysis was firstly performed to identify the ranking importance of variables (fungal OTUs and metabolites) for predicting OLS incidence (Breiman, 2001; Cutler et al., 2007). This analysis was set through machine learning algorithms, using the R RandomForest package (Cutler et al., 2007). For each tree grown on a bootstrap sample, the error rate for observations left out of the bootstrap sample was monitored. The predictor variables explained 74.1% and 85.2% of the variation in fungal OTUs and metabolites, respectively. The Gini coefficient indicates the variable contribution (importance) for OLS disease incidence. Spearman correlations and redundancy analyses (RDA) were then performed using the most important fungal OTUs and metabolites, which were pre-selected by the random forest analysis (Gini index >100). The Spearman correlations were computed using the R corrplot package (Wei et al., 2017), in order to check the correlation between fungal OTUs, metabolites and OLS incidence. RDA was performed using R vegan package (Oksanen et al., 2017), in order to find relationships among cultivars (“Cobrançosa”, “Madural”, “Verdeal Transmontana”), OLS disease incidence ranges (0-5%, 5-10%, 10-15%), fungal OTUs and secondary metabolites. One-way analysis of variance (ANOVA) was carried out with ‘anova’ function, to test significant differences between cultivars or OLS incidence groupings, previously obtained by RDA ordination based on fungal OTUs and metabolites.

## 3 Results

### 3.1 Differences on fungal communities and metabolite profiles among OLS incidence ranges and cultivars

Overall, 154 fungal operational taxonomic units (OTUs), 18 phenolic and 73 volatile compounds, were identified from all analyzed olive leaves (**Figures S1–S3**). Among the identified fungal genera, *Cladosporium*, *Alternaria* and *Fusarium* were the most frequently isolated, representing 35% of the total number of isolates. In what concerns metabolites, the phenolic compounds oleuropein, apigenin-7-O-glucoside, rutin and verbascoside, as well as the volatiles Z3-hexen-1-ol-acetate and Z3-hexen-1-ol, were the most abundant, accounting together for 78% and 82% of the total phenolic and volatile fraction, respectively.

In general, the number of both fungal OTUs and detected metabolites did not change significantly across the three levels of OLS disease incidence (**Figure S4**). In what concerns abundance, only the abundance of fungal isolates retrieved from the most OLS-susceptible cultivar (“Verdeal Transmontana”) exhibited a 2-fold significant increase (*p <*0.05) in trees with the highest OLS disease incidence. The comparison among cultivars, showed only differences on the number of volatile compound (**Figure S5**). Indeed, with an increase of OLS disease incidence, the levels of volatiles decreased significantly (*p*<0.05) in cv. “Cobrançosa”, while increased significantly (*p*<0.05) in cv. “Madural”.

The comparison between the endophytic and epiphytic communities across distinct OLS incidences showed differences in terms of diversity and abundance (**Figure S6**). With the highest OLS disease incidence, endophytic fungi displayed a greater increase in abundance (up to 1.4-fold, *p <*0.05) and richness (up to 1.1-fold, *p <*0.05) than epiphytic fungi. Regarding the epiphytic community, only a significant increase was detected for epiphytes abundance in trees with the highest OLS incidence (up to 1.3-fold, *p <*0.05).

**Figure 1:**
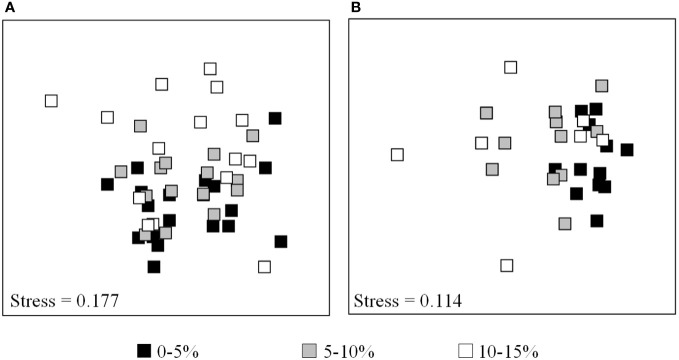
Non-metric multidimensional scaling (NMDS) plots of foliar fungal communities **(A)** and metabolite profiles **(B)** detected on olive trees displaying different levels of OLS disease incidence (0-5%, 5-10%, 10-15%). Clustering analysis was performed with Bray-Curtis distance. Kruskal’s stress values are displayed.

**Figure 2:**
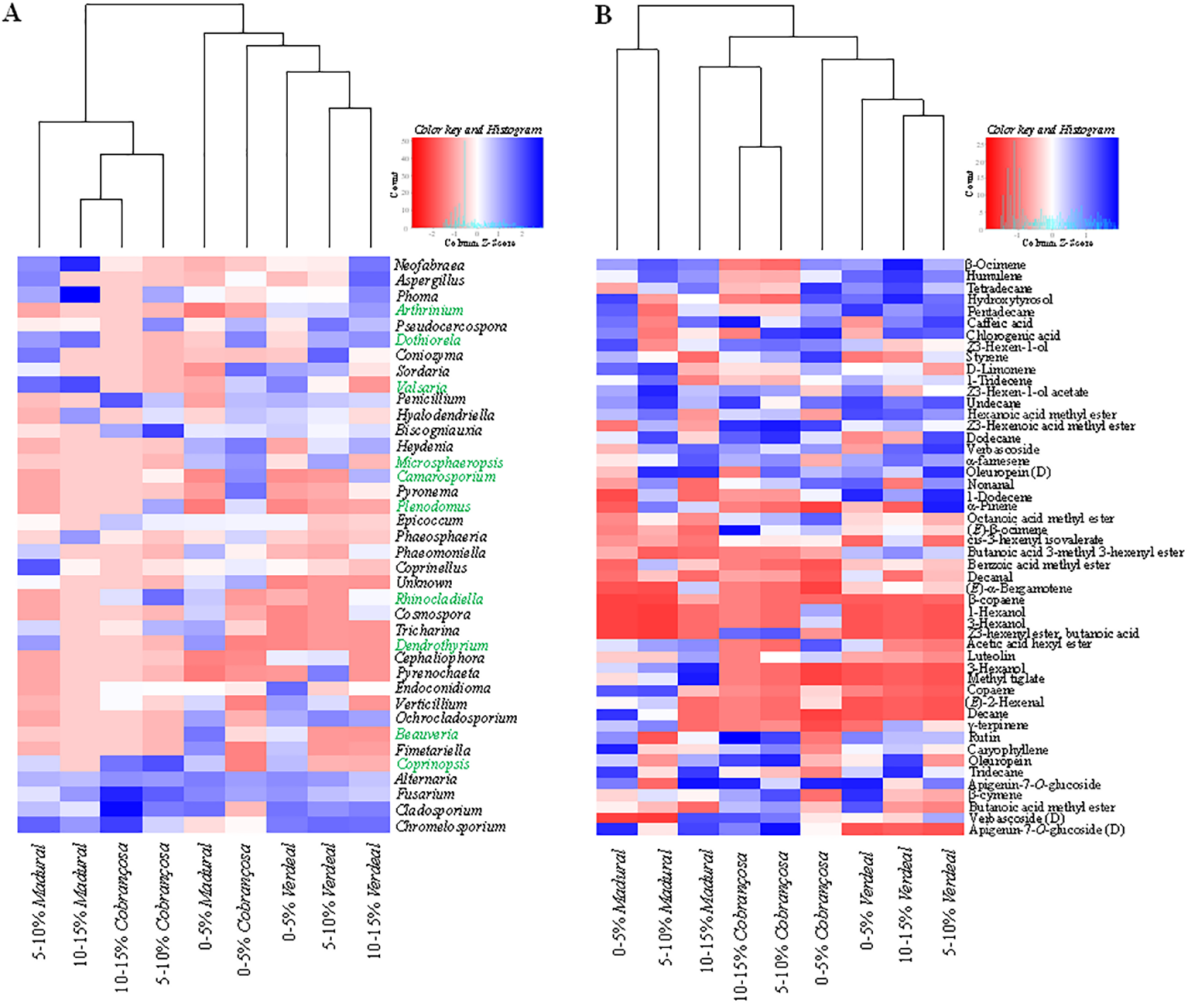
Variation of fungal communities **(A)** and metabolites profiles **(B)** in leaves of olive trees from distinct cultivars (“Cobrançosa”, “Madural” and “Verdeal Transmontana”), displaying different levels of OLS disease incidence (0-5%, 5-10% and 10-15%). Heat maps indicate differences in the relative abundances of the most abundant fungal OTUs and metabolites. The color-scale ranges from red z < -3 to blue z >3, indicating the abundance of fungal OTUs and metabolites. Fungal isolates exclusively found on the episphere (leaf surface) and endosphere (leaf interior) are shown in green and purple color, respectively.

The composition of fungal communities and metabolite profiles differs significantly among trees with distinct disease incidence levels (**Figure 1**; **Table S1**). Hierarchical cluster analysis based on the most abundant fungal OTUs also separated fungal communities into two main groups, corresponding to the communities found in trees with low OLS incidence and communities with higher incidence levels (**Figure 2A**). However, such separation was dependent upon the olive cultivar. Fungal communities from cv. “Verdeal Transmontana” clustered together, regardless of tree disease incidence. In contrast, the fungal composition from cvs. “Cobrançosa” and “Madural” differed when considering trees exhibiting high and low OLS incidence levels (ANOSIM, *R*=0.40, *p*=0.001). Accordingly, fungal communities from cv. “Verdeal Transmontana” were less distinct in trees with different OLS incidence levels (ANOSIM, *R*=0.33, *p*=0.002). Nevertheless, the ANOSIM analysis could still distinguish all three incidence levels in this cultivar (*R*=0.31, *p*=0.016). Differences on fungal community composition between cultivars were always lower in trees displaying the highest OLS disease incidence, which was particularly detected in cvs. Cobrançosa” and “Madural”. Indeed, the highest difference on fungal communities between cultivars was detected at the lowest OLS-disease incidence level (ANOSIM, *R*=0.72, *p*=0.001).

The metabolite profiles of trees from the three cultivars were distinct (ANOSIM, *R*=0.32, *p*=0.001), being these differences greater between cvs. “Madural” and “Verdeal Transmontana” (ANOSIM, *R*=0.35, *p*=0.001). Although less relevant than for fungal communities, leaf metabolite composition within each cultivar also varied with OLS-disease incidence, being the greatest differences observed among trees with the lowest and highest disease incidence levels (**Figure 2B**). This result was particularly observed for trees from cvs. “Madural” (ANOSIM, *R*=0.970, *p*=0.002) and “Cobrançosa” (ANOSIM, *R*=0.88, *p*=0.002), while cv. “Verdeal Transmontana” exhibited a similar metabolite composition among all trees (ANOSIM, *R*=0.125, *p*=0.079).

### 3.2 Relationship between host cultivar, foliar fungal community, metabolite profile and disease incidence

**Figure 3:**
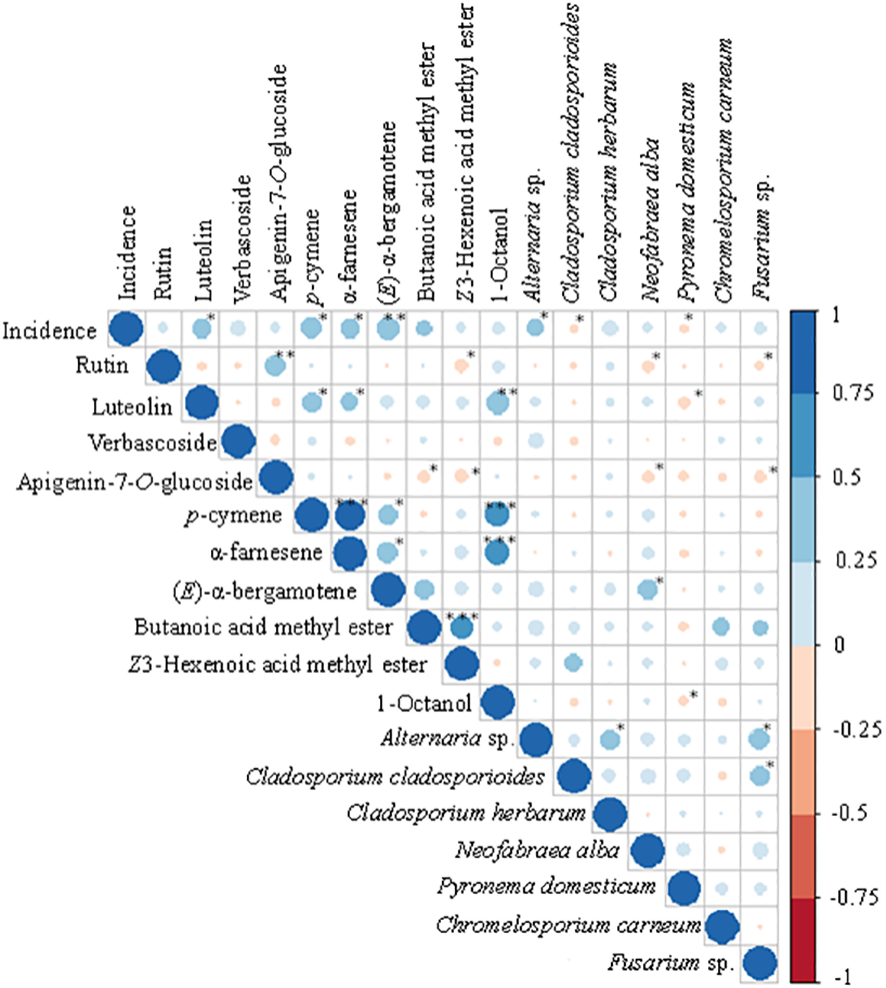
*Spearman* correlations between fungal OTUs abundance, metabolites concentration and OLS disease incidence. These correlations were only performed with variables (fungal OTUs and metabolites) preselected by the random forest analysis. Blue color represents positive correlations, while red color represents negative correlations. Circle size and color shading indicate correlation coefficient values. High coefficient values (maximum = 1) are represented by larger and darker circles, while smaller and lighter circles represent lower coefficient values (minimum = 0). Asterisks indicate statistically significant correlations at *p < 0.05, **p < 0.01 and ***p < 0.001.

One of the goals of this study was the identification of a set of fungal OTUs and metabolites that could explain differences in susceptibility of different olive tree cultivars to OLS disease. The complexity of this biological system, in which multiple interaction effects can occur between host plant, fungi, and metabolites, together with the large amount of microbial/metabolite produced data, increases the difficulty of this task. Thus, to more accurately predict such relationships, a random forest analysis was employed to select the most relevant variables (i.e. fungi/metabolite) for the prediction of OLS incidence. The random forest ranks the importance of input variables measured by a *Gini* coefficient value. A higher *Gini* coefficient value represents a greater variable importance (Cutler et al., 2007). Eight fungal OTUs and ten metabolites (four phenolics and six volatiles) were identified as the most important variables for determining OLS disease incidence (*Gini* coefficient > 100; **Figure S7**). For testing the association between fungi, metabolites and OLS disease incidence, the selected variables were then used to perform *Spearman* correlations (**Figure 3**). The results revealed that the volatiles (E)-α-bergamotene, α-farnesene and p-cymene, the phenolic luteolin, and the fungal OTUs *Alternaria* sp., exhibited significant positive correlations with disease incidence. In contrast, *Cladosporium cladosporioides* and *Pyronema domesticum* were negatively correlated with OLS disease incidence. Other fungal OTUs were also found to be either negatively or positively correlated with some metabolites, as well as with other fungal OTUs. Specific significant inter- and intra-group metabolites correlations also existed, being particularly observed a strong positive correlation between the volatiles α-farnesene and p-cymene, and between these two compounds and 1-octanol.

**Figure 4:**
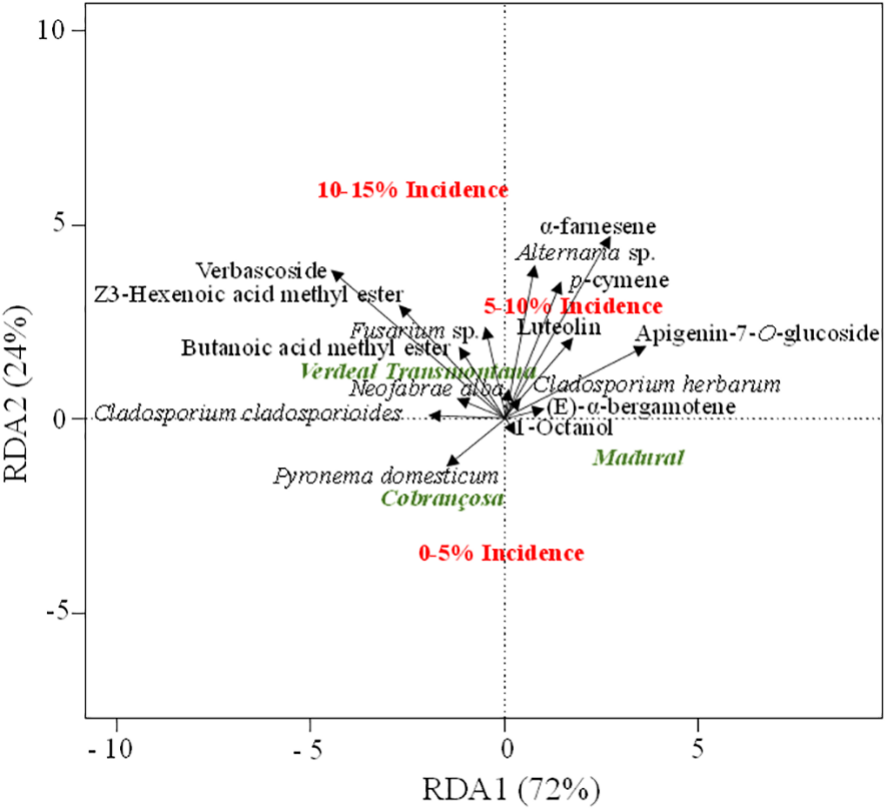
Redundancy analysis (RDA) ordination plot showing the association between the OLS disease incidence levels (0-5%, 5-10% and 10-15%), olive tree cultivars (“Cobrançosa”, “Madural” and “Verdeal Transmontana”), leaf fungal community and metabolites composition. This analysis was only performed with variables (fungal OTUs and metabolites) preselected by the random forest analysis. In RDA analysis, a positive correlation between two variables is expressed by relatively long vectors pointing approximately in the same direction, whereas a negative correlation is indicated by arrows pointing in opposite directions. The longer the variable decline, the stronger the relationship of that parameter with the olive cultivar/disease incidence. The percentage of variation is explained by each axis.

Since *Spearman* correlations only test the correlation between two variables, we have additionally performed a redundancy analysis (RDA). Beyond a simple ordination among the variables (unconstrained ordination, as in PCA), the explanatory variables are used in this analysis to test their predictive power on the multidimensional variable space (constrained ordination). The final outcome expresses how much of the variance in the set of response variables (fungal OTUs and metabolites) is explained by the set of explanatory variables (host cultivar and disease incidence level). Note that the order of explanatory to predictor variables was inverted relatively to the random forest analysis. Here, the question is how well OLS disease incidence levels or olive cultivars discriminate among metabolites or components of the fungal community. As for the previous *Spearman* correlations, only the most important variables preselected by the random forest analysis were used in RDA. OLS disease incidence ranges (*p <*0.01) and olive cultivars (*p <*0.01) were clearly discriminated among foliar fungal community and metabolite profiles (**Figure 4**). Several correlations were also detected between metabolites, fungal OTUs, host cultivars, and OLS disease incidence. The strength of such correlations was assessed by the arrow length and angle between arrows and axes. The lowest OLS disease incidence range is more closely associated to cv. “Cobrançosa”, and to the presence of *P. domesticum* on leaves. In contrast, higher OLS disease incidence is more associated to cv. “Verdeal Transmontana”. The segregation of trees with higher OLS disease incidence is based on their metabolite and fungal profiles. Disease incidence of 5-10% was mostly related with the metabolites α-farnesene, p-cymene, apigenin-7-O-glucoside and to the presence of *Alternaria* sp.; while the highest disease incidence (10-15%) was more closely associated to verbascoside, Z3-hexenoic acid, methyl ester, butanoic acid, methyl ester, and to the presence of *Fusarium* sp.

## 4 Discussion

To the best of our knowledge, the present study is the first work to address the relationship between host plant, foliar fungal communities, metabolic profiles and plant disease incidence under field conditions. We attempted to determine whether differences in susceptibility of different olive tree cultivars to OLS disease is linked to fungal communities and/or metabolite composition of host plant leaves.

Our results underline the importance of fungal communities inhabiting the leaves of each olive tree as possible determinants of disease incidence. Indeed, several significant correlations occurred between the abundance of specific fungi (*Alternaria* sp., *C. cladosporioides* or *P. domesticum*) and the incidence of OLS disease. Moreover, the relation between fungal composition and disease incidence was found to be dependent on host cultivar (and thereby on genotype susceptibility). When analyzing the three levels of OLS disease incidence, a greater variation on the foliar fungal composition was detected in the OLS-tolerant cv. “Cobrançosa*”*, in comparison with the susceptible cv. “Verdeal Transmontana*”*. We hypothesize that fungal community changes could affect OLS disease incidence, probably due to the role of endophytic/epiphytic fungi that could act as biocontrol agents for olive diseases (Poveda and Baptista, 2021). A reduction on the abundance of those fungi able to provide host plant protection against OLS disease could determine higher disease incidence in the most tolerant cultivar. Contrasting with what occurs in the tolerant cultivar, the pathogen could be more adapted to the leaf fungal community on the most OLS-susceptible cultivar (cv. “Verdeal Transmontana*”*).

The pathogen *V. oleaginea* itself is able to alter the fungal community of leaves during disease development. Accordingly, those trees displaying the highest OLS disease incidence exhibit a more similar foliar fungal community composition, regardless of the olive tree cultivar considered. The results from the present study are in line with the now accepted idea that disease development and progression depend on pathogen adaptation to the new environment, as well as on the interactions outcomes established with other microorganisms in the shared niche (McNally and Brown, 2015). Although microbiota studies can help to understand the role of other fungi for the development of olive diseases, it would be difficult to determine whether the reported changes are really due to the pathogen itself or are only a result from disease development. Taking this into consideration, changes in the fungal microbiota of olive in the presence of different diseases, as those caused by *Xylella fastidiosa* (Giampetruzzi et al., 2020), *Pseudomonas savastanoi* pv. *savastanoi* (Gomes et al., 2019), *Colletotrichum* sp. (Martins et al., 2021), and even *V. oleaginea* (Varanda et al., 2019), have been studied. For example, when studying OLS disease, Varanda et al. (2019) revealed a relation of OLS disease and the abundance of specific isolates, such as *Chalara* sp. and *Foliophoma* sp., while the absence of disease was related to the presence of *Alternaria* sp. and *Epicoccum* sp. isolates. These results contradict the findings from the present work, in which the presence of *Alternaria* sp. was strongly related to the development of the disease. These results suggest that other factors could be affecting plant disease development as well.

In the present study, leaf volatile emissions changed both quantitatively and qualitatively in leaves from trees exhibiting different incidences of OLS disease. Detected variations were different according to the host cultivar, suggesting that volatile compounds can probably contribute to plant OLS-resistance/tolerance. As far as we know, this is the first time in which such differences were detected according to the cultivar susceptibility to disease, leaving us to speculate on the underlying mechanism. Differences on cultivar susceptibility can be caused by multiple factors, including the activation of different plant defense pathways. Indeed, in a meta‐analysis about induced plant volatiles, the effects of pathogenic infections caused by distinct fungi were attributed to differences in the induced defense pathways (Ameye et al., 2018). Curiously, on the most OLS-tolerant cultivar, a suppression rather than an induction of volatile emissions was observed in trees with increasing levels of disease incidence. Similar results were obtained following pathogen attacks in maize and potato plants (Seidl-Adams et al., 2015; Moreira et al., 2021). The reduction on volatile emissions has been associated with enhanced defense responses, suggesting that volatiles may also act as disease suppressors (Erb, 2018). However, little is known about such volatile capacity and mechanisms involved in the process (Erb, 2018). In the present work, specific volatile compounds (i.e. α-farnesene, p-cymene and 1-octanol) were found to be positively correlated with each other and with OLS incidence, suggesting that they may be integrated in a specific pathway and contribute to a higher OLS incidence. Given the capacity of volatiles to regulate different signaling cascades involved in plant defense, the integration of these volatile compounds through a signaling crosstalk is likely to occur (Erb, 2018).

The phenolic composition of olive tree leaves also changed with OLS disease incidence levels, displaying a variable pattern that depends on the cultivar. As for volatile compounds, the observed differences on phenolics might reflect the variation of olive tree cultivars on their susceptibility to disease. A relation between phenolic composition and susceptibility to infection was previously found in Norway spruce when attacked by the needle bladder rust (Ganthaler et al., 2017), or in maize after infection with *Fusarium verticillioides* (Bernardi et al., 2018). The possible contribution of phenolic compounds to OLS resistance/tolerance of host cultivar was further reinforced by the positive correlation found between some phenolic compounds (*i.e.* luteolin, rutin, verbascoside and apigenin-7-O-glucoside) and OLS disease incidence.

Previous works on plant defense responses to pathogen attacks mainly used reductionist approaches, by focusing on host plant protection conferred by either fungal (Collinge et al., 2022) or plant secondary metabolites (Zaynab et al., 2018). In the present study, disease incidence was interlinked for the first time to host cultivar, to fungal communities inhabiting leaves and to leaf metabolite composition. Different olive tree cultivars, grown in the same field, exhibited distinct fungal communities on their leaves and displayed diverse leaf metabolite compositions. Thus, host cultivar appears to affect, not only leaf fungal composition, but also metabolite profiles. Moreover, the interaction effects between fungi and metabolite compounds could also play an important role on the composition of each other. Accordingly, changes on fungal and metabolite composition in leaf samples from trees with different incidence levels of OLS disease revealed a similar trend, suggesting a possible link between fungi and metabolites. This relationship is further reinforced by the significant correlations found between certain fungal OTUs and metabolites. Although further analysis is required, we hypothesize that fungal communities residing in olive leaves could influence the metabolites of host plant, as previously observed by *Trichoderma* endophytes (Marra et al., 2020; Dini et al., 2021). Reciprocally, leaf metabolites could also affect fungal communities on olive leaves, as previously suggested for other plant species (Zambell and White, 2017).

A strength of our work is the identification of fungal OTUs and secondary metabolites strongly associated with OLS disease incidence. The lowest level of OLS incidence, which was found to be associated to the most OLS-tolerant cultivar, was linked to the presence of *P. domesticum* that appears to suppress OLS disease. This possibility is worth investigating further in the future. Although *P. domesticum* has already been described to colonize the inner tissues of other plant species (Ghasemi et al., 2019), information about their role in conferring host plant protection against biotic stress is completely lacking. The highest level of OLS incidence, which was associated to the most OLS-susceptible cultivar (cv. “Verdeal Transmontana*”*), was found to be positively correlated with various fungal OTUs and metabolites. Among the fungal taxa positively correlated with OLS incidence, both *Alternaria* sp. and *Fusarium* sp. have been extensively described as important plant pathogens causing numerous diseases in several plant species (Hernandez-Escribano et al., 2018; Wei et al., 2018). In what concerns olive tree, only few reports described their capacity to infect olive fruits, causing fruit-rot (Moral et al., 2008; Trapero-Casas et al., 2009). Both genera have been described as making part of synergistic pathogen-pathogen interactions that often lead to increased disease severity (Lamichhane and Venturi, 2015). Thus, both fungi are likely to play a similar role in our pathosystem.

Besides the fungal role on OLS disease development, the positive correlation of specific secondary metabolites with OLS incidence could also implicate them on OLS disease development or as part of plant defense responses. Among the positively correlated metabolites, both α-farnesene and p-cymene seem to be the most important volatiles produced in leaves from trees with higher OLS disease incidence. Both sesquiterpenes have been described as important players on plant defenses against pathogen attacks (Runyon et al., 2020; Lemaitre-Guillier et al., 2021), suggesting a potential defensive role. In a similar way, other phenolic compounds, apigenin-7-O-glucoside and verbascoside, could play a role on OLS plant responses, since their levels have been previously described to increase after pathogen infection (Markakis et al., 2010; Schmidt et al., 2015). In addition, other phenolics (*e.g.* flavonoids and cinnamic acid derivatives) and volatile (*e.g.* ester) compounds were also positively correlated with OLS disease incidence, although without significance. Therefore, the role of positively correlated metabolites with OLS disease incidence is more likely to be part of plant defense responses to pathogen attack.

In conclusion, both fungal communities and metabolite compositions, in association with plant genotype, seem to play an important role on OLS disease incidence. The OLS-tolerant cv. “Cobrançosa” displayed greater variation in fungal and metabolite assemblages among trees with different OLS incidence, when compared to OLS-susceptible cv. “Verdeal Transmontana”. Thus, differences on cultivar OLS-susceptibility are likely to be related with leaf fungal composition, metabolites (both phenolic and volatile compounds), and a combination of both. The complex interactions occurring between the host plant (cultivar), fungi and metabolite composition will influence the OLS disease incidence. Our work identified several key fungi and metabolites that could play an important role in the susceptibility/tolerance of cultivars to OLS disease. In this regard, future studies on the interactions of *Pyronema domesticum* with olive tree and *V. oleaginea* pathogen could provide functional roles of this fungus in host susceptibility/resistance to OLS disease.

## Data availability statement

The datasets presented in this study can be found in online repositories. The names of the repository/repositories and accession number(s) can be found in the article/**Supplementary Material**.

## Author contributions

PB and JP designed the experiments and together with TL-N supervised the study and revised the manuscript. TG performed most of the experiments, analyzed the data and drafted the manuscript. JM-L assisted with data analysis and together with JP revised the manuscript. All authors contributed to the article and approved the submitted version.
