# Supplementary material for: Deciphering plant health status: The link between secondary metabolites, fungal community and disease incidence in olive tree
Source: Front Plant Sci. 2023 Mar 22;14:1048762. doi: 10.3389/fpls.2023.1048762 (PMC10073708; doi:10.3389/fpls.2023.1048762)
Supplement: Supplementary file 1 [file DataSheet_1.docx]

Supplementary Material

# Supplementary Figures and Tables

## Supplementary Figures

**
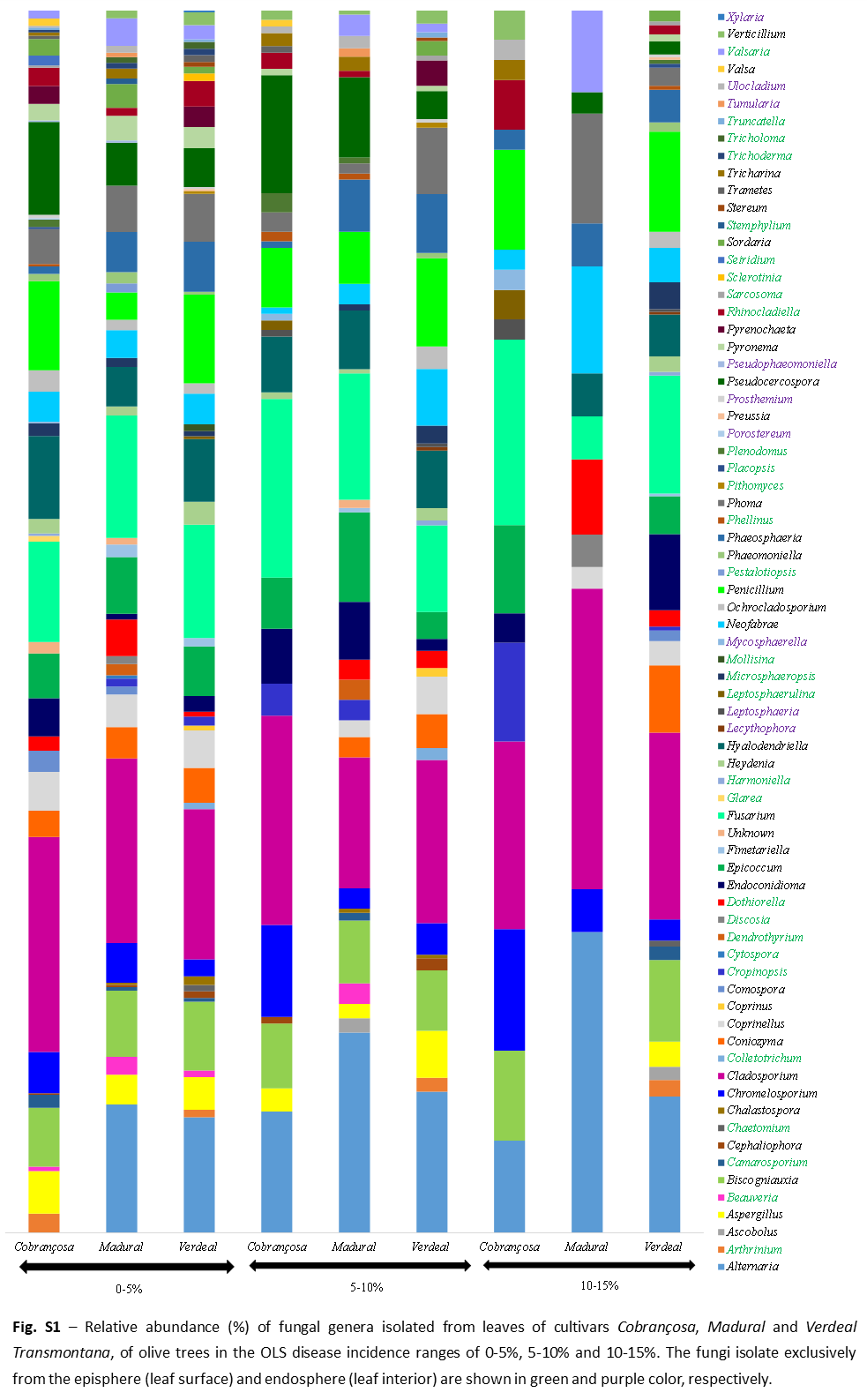
**

**Supplementary Figure 1.** Relative abundance (%) of fungal genera isolated from leaves of cultivars *Cobrançosa*, *Madural* and *Verdeal Transmontana*, of olive trees in the OLS disease incidence ranges of 0-5%, 5-10% and 10-15%. The fungi isolated exclusively from the episphere (leaf surface) and endosphere (leaf interior) are shown in green and purple color, respectively.


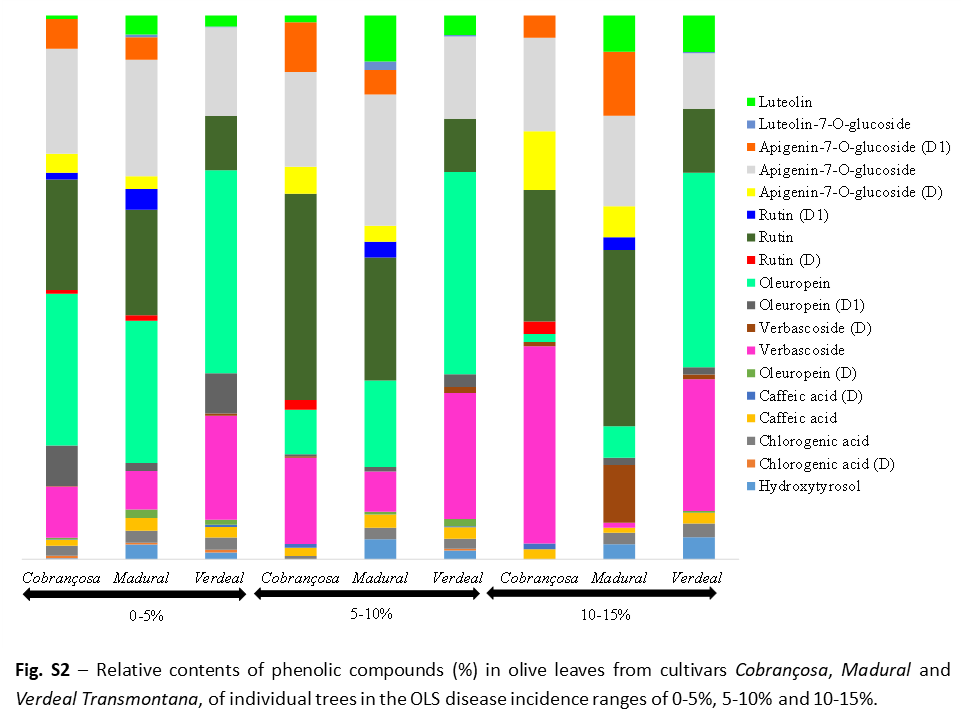


**Supplementary Figure 2.** Relative contents of phenolic compounds (%) in olive leaves from cultivars *Cobrançosa*, *Madural* and *Verdeal Transmontana*, of individual trees in the OLS disease incidence ranges of 0-5%, 5-10% and 10-15%.


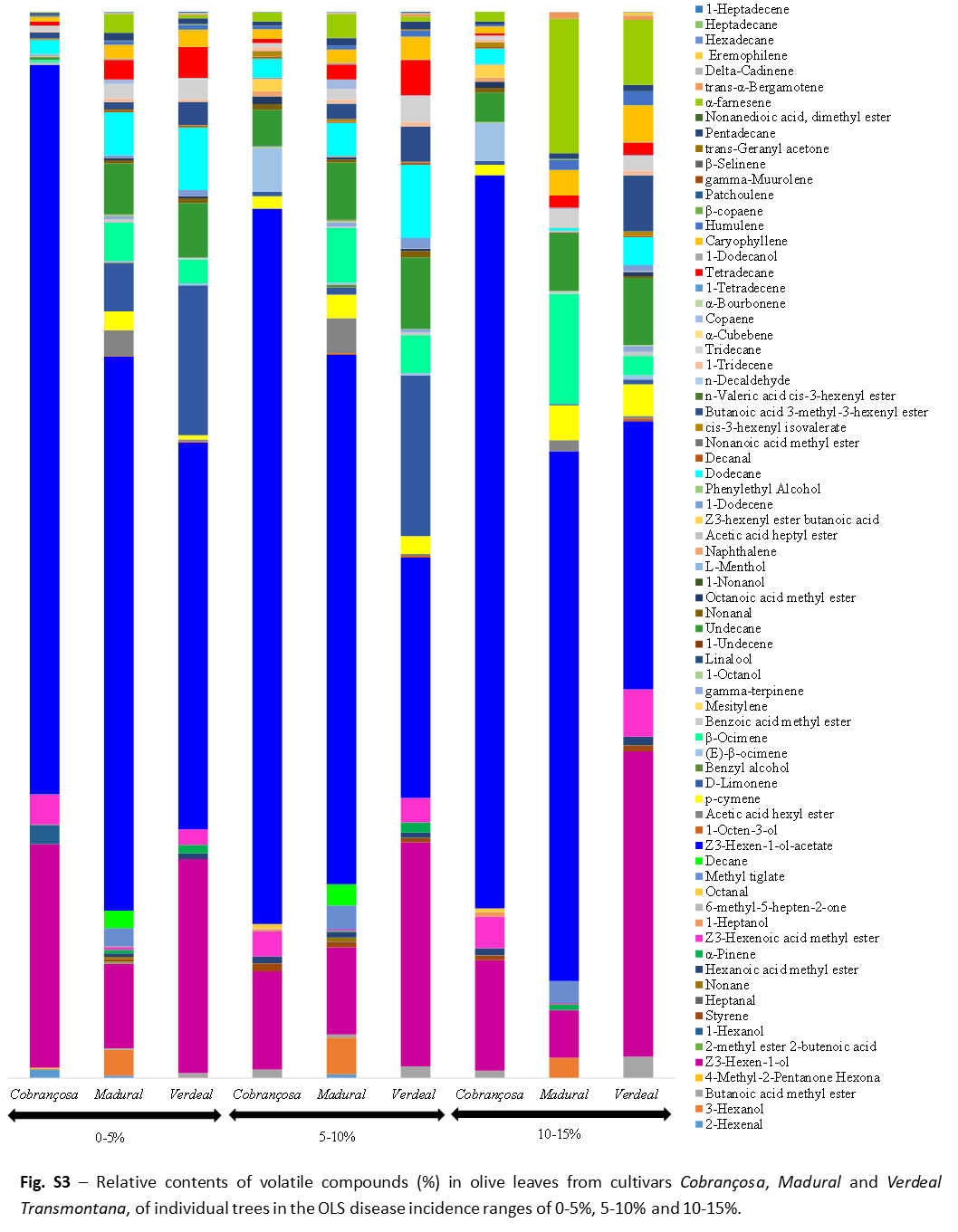


**Supplementary Figure 3.** Relative contents of volatile compounds (%) in olive leaves from cultivars *Cobrançosa*, *Madural* and *Verdeal Transmontana*, of individual trees in the OLS disease incidence ranges of 0-5%, 5-10% and 10-15%.


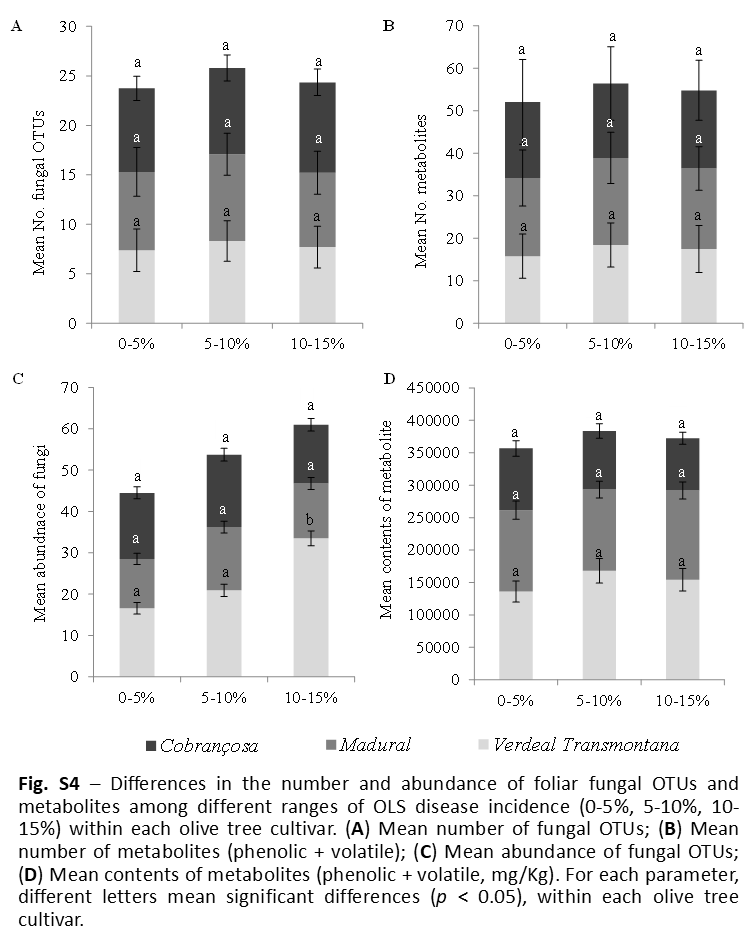


**Supplementary Figure 4.** Differences in the number and abundance of foliar fungal OTUs and metabolites, among different ranges of OLS disease incidence (0-5%, 5-10%, 10-15%) within each olive tree cultivar (*Cobrançosa*, *Madural* and *Verdeal Transmontana*). (**A**) Mean number of fungal OTUs; (**B**) Mean number of metabolites (phenolic + volatile); (**C**) Mean abundance of fungal OTUs (log CFU/cm^2^); (**D**) Mean contents of metabolites (phenolic + volatile, mg/kg). For each parameter, different letters mean significant differences (p < 0.05), within each olive tree cultivar.


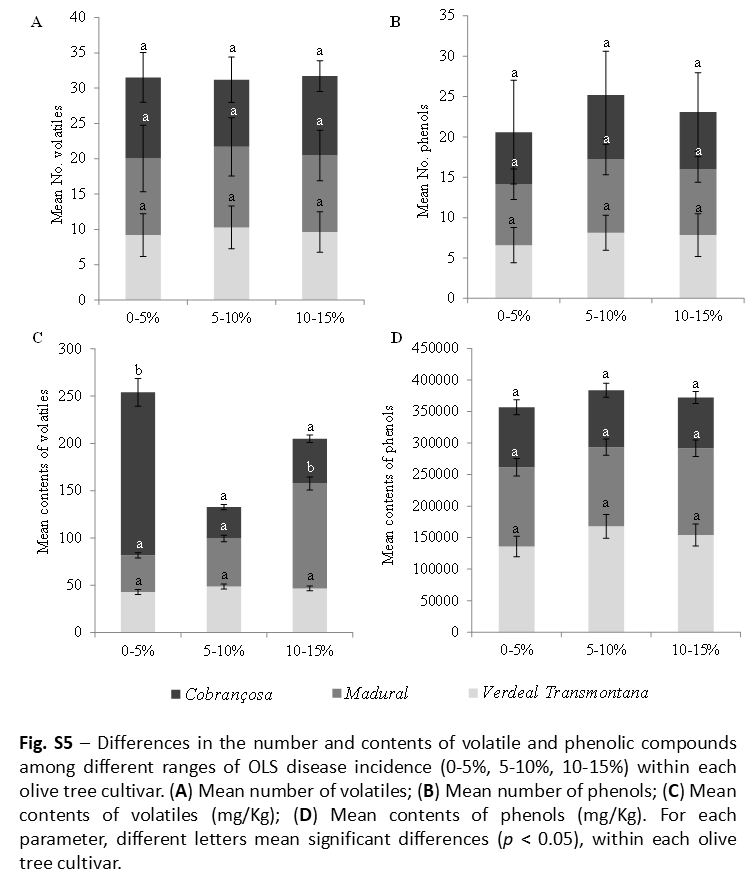


**Supplementary Figure 5.** Differences in the number and contents of volatile and phenolic compounds among different ranges of OLS disease incidence (0-5%, 5-10%, 10-15%), within each olive tree cultivar (*Cobrançosa*, *Madural* and *Verdeal Transmontana*). (**A**) Mean number of volatiles; (**B**) Mean number of phenolic compounds; (**C**) Mean contents of volatiles (mg/Kg); (**D**) Mean contents of phenolic compounds (mg/Kg). For each parameter, different letters mean significant differences (p< 0.05), within each olive tree cultivar.


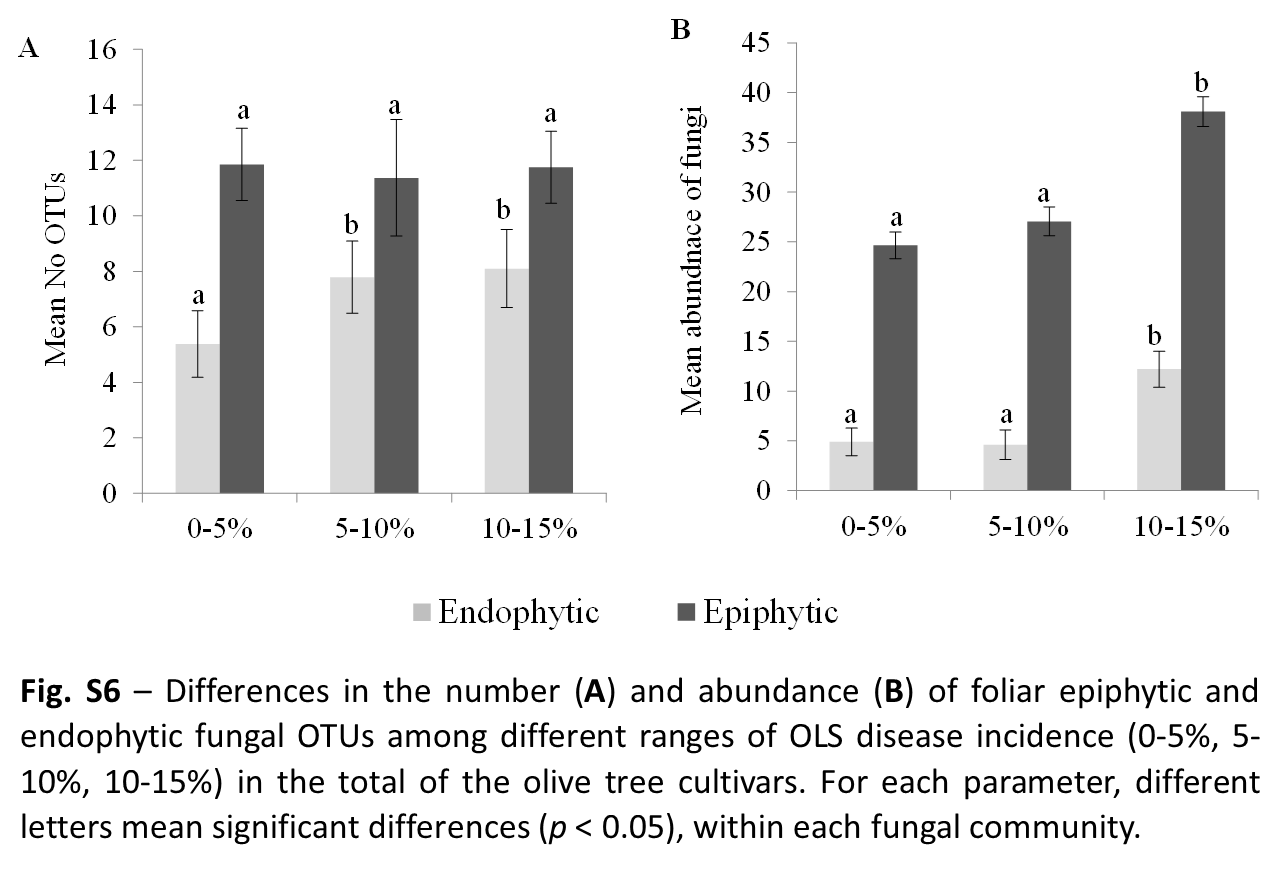


**Supplementary Figure 6.** Differences in the number (**A**) and abundance (**B**, CFU/cm^2^) of foliar epiphytic and endophytic fungal OTUs among different ranges of OLS disease incidence (0-5%, 5-10%, 10-15%) in the total of the olive tree cultivars. For each parameter, different letters mean significant differences (p< 0.05), within each fungal community.


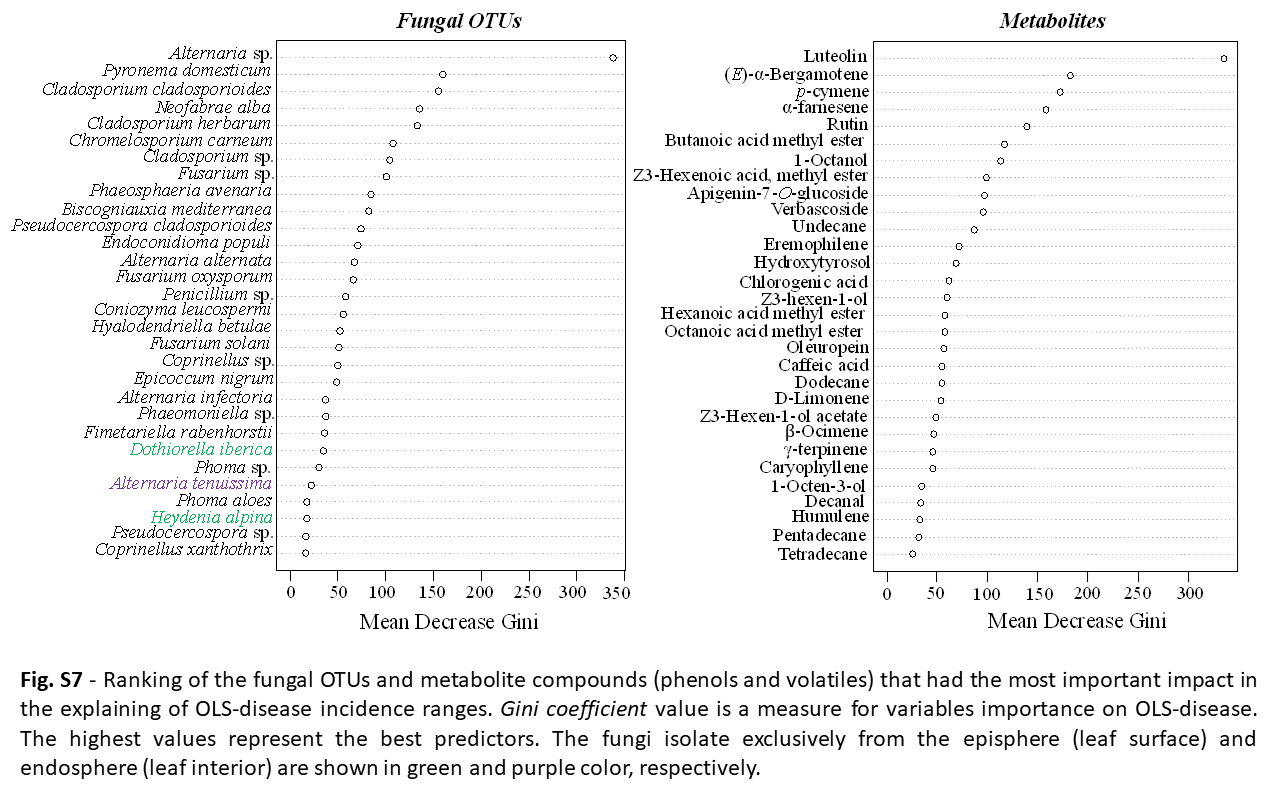


**Supplementary Figure 7.** Ranking of the fungal OTUs and metabolite compounds (phenols and volatiles) that had the most important impact in the explaining OLS-disease incidence ranges. *Gini coefficient* value is a measure for variables importance on OLS-disease. The highest values represent the best predictors. The fungi isolated exclusively from the episphere (leaf surface) and endosphere (leaf interior) are shown in green and purple color, respectively.

## Supplementary Tables

**Supplementary Table 1.** Analysis of similarity (ANOSIM), based on Bray-Curtis distance, of the foliar fungal communities and metabolites composition between different ranges of OLS disease incidence (0-5%, 5-10%, 10-15%) in the total of olive tree cultivars. Included are the R-statistics (R) and *p*-values.

| OLS disease incidence ranges comparison | Fungal community | |  | Metabolites | |
| --- | --- | --- | --- | --- | --- |
|  | R | *p* |  | R | *p* |
| 0-5% *vs.* 5-10% | 0.35 | 0.001 |  | 0.33 | 0.001 |
| 5-10% *vs*. 10-15% | 0.39 | 0.001 |  | 0.16 | 0.021 |
| 0-5% *vs.* 10-15% | 0.41 | 0.001 |  | 0.55 | 0.001 |

**Supplementary Table 2.** Analysis of similarity (ANOSIM), based on Bray-Curtis distance, of the foliar epiphytic and endophytic fungal communities composition between different ranges of OLS disease incidence (0-5%, 5-10%, 10-15%) in the total of olive tree cultivars. Included are the R-statistics (R) and *p*-values.

| OLS disease incidence ranges comparison | Epiphytic community | |  | Endophytic community | |
| --- | --- | --- | --- | --- | --- |
|  | R | *p* |  | R | *p* |
| 0-5% *vs.* 5-10% | 0.33 | 0.006 |  | 0.39 | 0.002 |
| 5-10% *vs*. 10-15% | 0.35 | 0.001 |  | 0.16 | 0.018 |
| 0-5% *vs.* 10-15% | 0.37 | 0.017 |  | 0.44 | 0.001 |
